# Renal allograft survival rates in kidneys initially declined for paediatric transplantation

**DOI:** 10.1007/s00467-018-3969-4

**Published:** 2018-05-28

**Authors:** Matko Marlais, Laura Pankhurst, Kate Martin, Lisa Mumford, E. Jane Tizard, Stephen D. Marks

**Affiliations:** 1 University College London Great Ormond Street Institute of Child Health, London, UK; 2 NHS Blood and Transplant, Bristol, UK; 3 University Hospitals Bristol NHS Foundation Trust, Bristol, UK; 4 Department of Paediatric Nephrology, Great Ormond Street Hospital for Children NHS Foundation Trust, Great Ormond Street, London, WC1N 3JH UK

**Keywords:** Paediatric nephrology, Renal transplantation, Transplant outcomes

## Abstract

**Background:**

The outcome of organs which have been declined for paediatric recipients is not known. This study aimed to determine the outcome of kidneys initially declined for paediatric recipients and establish renal allograft survival in kidneys that were eventually transplanted.

**Methods:**

Data were obtained from the UK Transplant Registry for all donation after brain death (DBD) kidneys offered and declined to paediatric recipients (< 18 years) in the UK from 2009 to 2014.

**Results:**

Eighty-two percent (503/615) of kidneys initially declined for paediatric transplantation were eventually transplanted, 7% (46/615) of kidneys went to paediatric recipients and 62% (384/615) of kidneys went to adult (kidney only) recipients. The remainder were used for multiple organ transplants. In the 46 kidneys that went to paediatric recipients, 1 and 3-year renal allograft survivals were 89% (95% CI 75.8–95.3%) and 82% (95% CI 67.1–90.6%), respectively. In the 384 kidneys given to adult kidney-only recipients, 1 and 3-year renal allograft survivals were 96% (95% CI 93.5–97.6%) and 94% (95% CI 90.7–96.1%), respectively. Eighty-four percent of the 204 children who initially had an offer declined on their behalf were eventually transplanted and have a functioning graft at a median 3-year follow-up.

**Conclusions:**

This study reports acceptable short-term renal allograft survival in kidneys that were initially declined for paediatric recipients and subsequently transplanted. Evidence-based guidelines are required to ensure that the most appropriate kidneys are selected for paediatric recipients.

## Introduction

Pre-emptive renal transplantation is the gold standard renal replacement therapy for children with end-stage kidney disease (ESKD). There is a significant body of adult data supporting the advantages of renal transplantation over dialysis, including improved survival and quality of life [1–3]. The deleterious effects of long-term sub-optimal dialysis in children are even more pronounced, including additional negative effects on growth and development. The priority for many paediatric nephrologists is to perform pre-emptive, in most cases live-related, renal transplantation for children, but where a living donor is not available, a child may be listed for a donation after brain death (DBD) and/or donation after cardiac death (DCD) kidney transplant. In the UK, paediatric patients receive the highest priority for deceased donor kidney offers (at each HLA match level), as well as receiving additional priority points if their HLA match is not favourable.

Transplant networks in different countries vary in their organ procurement and allocation methods, but when a DBD kidney is matched to a paediatric recipient, the child’s nephrologist and transplant surgeon make the decision whether to accept this kidney for this child. This decision is a time-critical one which needs to be made quickly [4]. Paediatric transplant centres in the UK have 45 min to respond to a DBD kidney offer. This is a challenging decision where the potential benefits of transplantation need to outweigh the risks. The risks in transplantation include a variety of donor and recipient factors [5–7], and sometimes an offer of a kidney is declined for a paediatric recipient. Whilst there are a number of possible reasons for decline, the most frequent reasons for decline are not well described in the literature.

Kidneys which are declined for donor factors, such as poor donor health or cause of death, may not be suitable for transplantation in any recipient. Other kidneys which are declined may subsequently be offered to another recipient (adult or child) and may be successfully transplanted. There are no reports in the literature which systematically evaluate the outcome in kidneys which have been declined for paediatric transplantation.

The outcome for children who have a kidney transplant declined on their behalf is also poorly understood, and further evidence is required to help clinicians decide whether a child may benefit from accepting a kidney sooner rather than waiting for a kidney of potentially higher quality or appropriateness.

The primary aim of this study was to determine the outcome of kidneys which were declined for paediatric recipients and establish renal allograft survival in those kidneys that were eventually transplanted. Secondary aims were to investigate the outcomes for children who had a kidney declined on their behalf and to investigate the most common reasons for declining a kidney for a paediatric recipient.

## Materials and methods

This study was conducted into two parts, a retrospective analysis of national data from the UK Transplant Registry and a prospective observational study.

### Retrospective analysis of kidney and patient outcomes after a declined kidney transplant offer for a paediatric recipient

Data were obtained from the UK Transplant Registry for all DBD kidneys offered and declined for paediatric recipients under 18 years of age in the UK from 1 January 2009 to 31 December 2014. Data on the total number of paediatric transplants and the number of children on the transplant waiting list at 1 January of each year was also collected.

### Outcome of kidneys that were initially declined for paediatric transplantation

We performed a retrospective analysis on the final outcome of kidneys declined on behalf of paediatric recipients during this time period, categorising kidneys as not transplanted or transplanted, and separating the latter category into the type of transplant. One and 3-year patient and renal allograft survival were calculated where the kidney was eventually transplanted. Data were separately analysed for those kidneys declined for donor reasons or inappropriate size for paediatric recipients.

### Outcome in paediatric patients for whom a kidney was offered and declined

The outcomes for the patients for whom the kidney was offered and declined were examined. Children were categorised into those who received a transplant and those who were still waiting for a transplant, and then sub-divided according to this. For those children who received a kidney transplant after their declined offer, the time from first declined offer to transplantation was calculated. Those children who had an initial decline for donor health or size mismatch were analysed separately, in order to eliminate those where the kidney decline was purely due to recipient reasons (e.g. recipient acutely unwell at the time of kidney offer). This was felt to give the clearest data representing organs that were declined for donor reasons and could be transplanted into other recipients.

### Prospective study of reasons for decline of a kidney for a paediatric recipient

For two separate years (2011 and 2014), a prospective observational study was conducted. During these years (from 1 January to 31 December in each year), the paediatric renal transplant centre that had declined an organ for a paediatric recipient received a supplemental data collection form to complete which requested additional information on the reason for declining the kidney for the paediatric recipient, with both generic reasons for decline and the option of free text comments as required (examples of generic reasons included; donor unsuitable due to cause of death, meningococcemia with < 48 h of treatment, no staff/theatre time, etc.). In addition to the routine data held by the UK Transplant Registry on declined offers, donor and recipient characteristics and anthropometrics were analysed.

### Statistical analysis

Results were analysed using summary statistics and 95% confidence intervals calculated as appropriate. The Kaplan-Meier method was used to estimate univariate post-transplant patient and renal allograft survival separately for all cases where the kidney was subsequently transplanted after being declined for a paediatric patient. Death censored renal allograft survival was defined as time from renal transplantation to renal allograft failure. Patient survival was defined as time from transplant to patient death censoring for patients still alive at the time of analysis.

Data were fully anonymised and ethical principles adhered to throughout the study; external ethical review was not required. Statistical analyses were performed in SAS version 9.2 (SAS Institute Inc., Cary, North Carolina, USA). All statistical tests are two-tailed and a *p* value of < 0.05 was considered statistically significant.

## Results

### Retrospective analysis of kidney and patient outcomes after a declined kidney transplant offer for a paediatric recipient

**Fig. 1:**
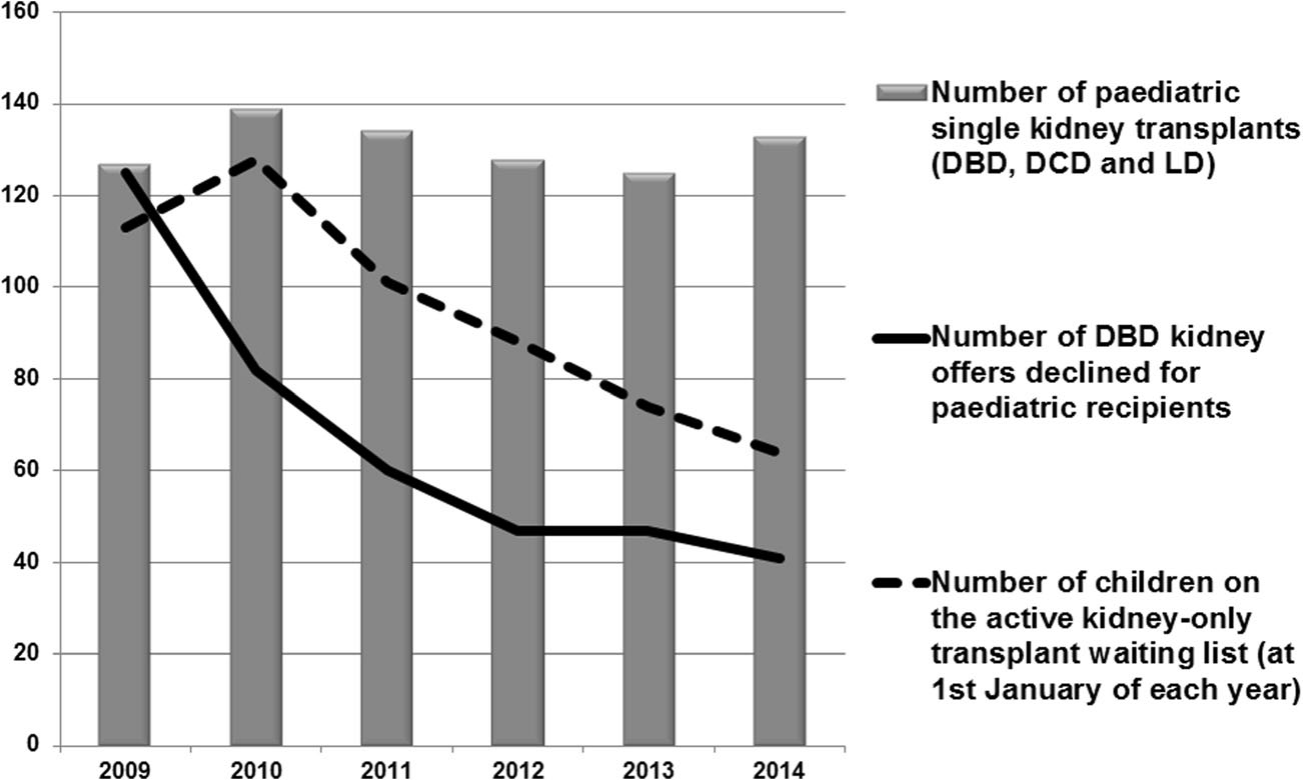
Total number of kidney offers declined for paediatric recipients from 2009 to 2014, with the total number of transplants in children and number of children on the active kidney transplant waiting list (all values are per calendar year across the UK). Type of kidney transplants: *DBD* donation after brain death, *DCD* donation after cardiac death, *LD* living donor

The total number of declined paediatric kidney offers per year reduced during the study as did the total number of children on the active kidney transplant waiting list (Fig. 1).

### Outcome of kidneys that were initially declined for paediatric transplantation

**Fig. 2:**
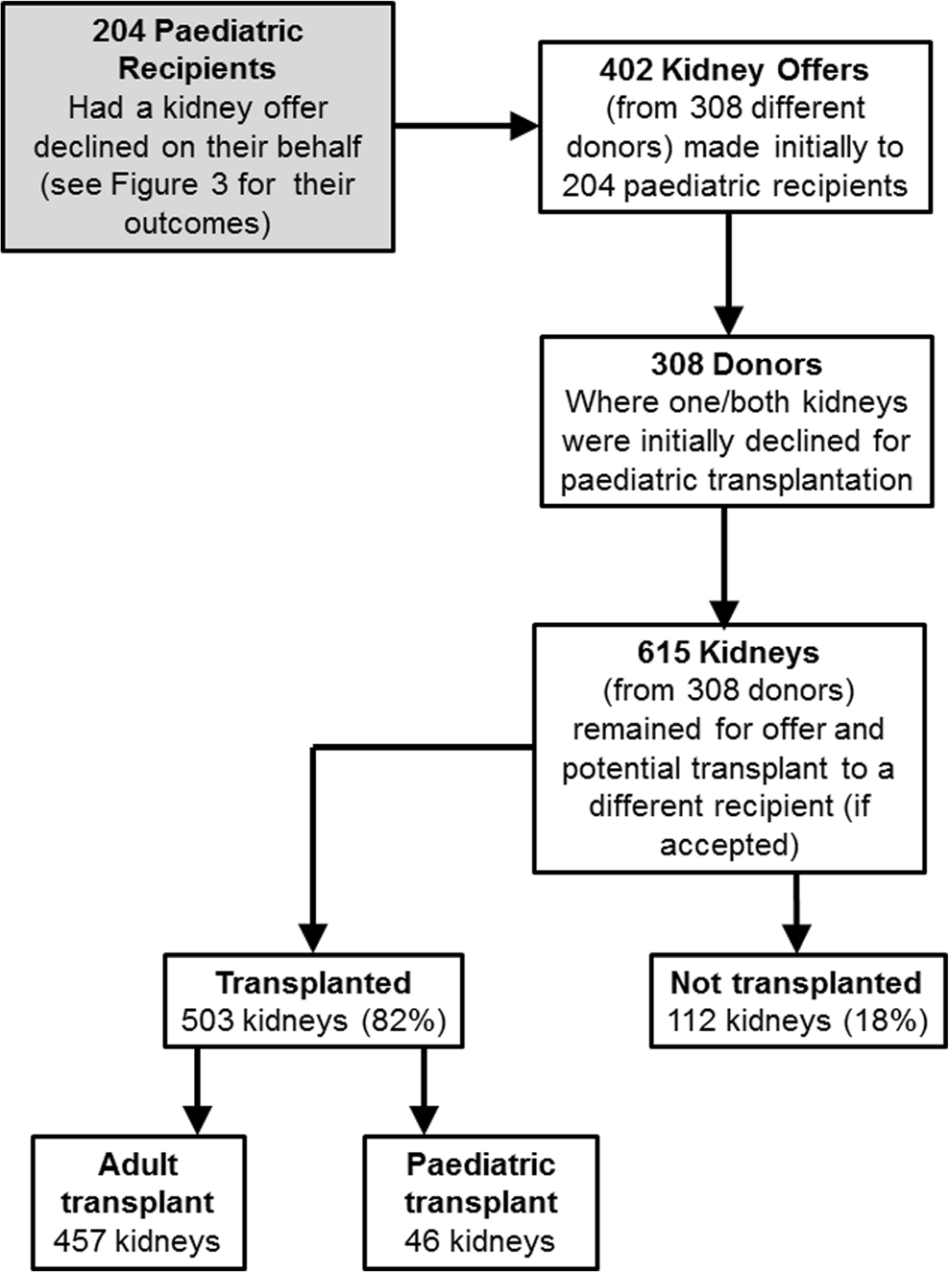
Flow diagram showing the number of recipients, donors and kidneys involved in each stage of this study, considering kidney offers declined for paediatric recipients in the UK from 2009 to 2014. The outcomes of kidneys initially declined for paediatric recipients are also shown

Four hundred and two DBD kidney offers from 308 different donors were declined for 204 different paediatric recipients from 2009 to 2014 in the UK. Some donor kidneys were offered to multiple children on the waiting list as some were declined more than once. Two hundred and four children had kidneys declined on their behalf, and some children had multiple kidney declines. Six hundred and fifteen kidneys were available for transplantation from the 308 donors who were declined for paediatric transplantation. We analysed the outcome for both kidneys (where the donor had two kidneys) even if only one of the kidneys was declined for paediatric transplantation. Therefore, the analysis of outcomes for 615 kidneys exceeds the number of declined offers (402); see Fig. 2.

Of 615 kidneys from 308 donors declined for paediatric recipients during the 6 years of the study, 7% (46) of 615 kidneys went to paediatric recipients (kidney-only), 74% (457) of 615 kidneys went to adult recipients (62% kidney-only and 10% simultaneous pancreas and kidney (Table 1). The remaining 2% were either double kidney or another multi-organ transplant involving a kidney; the numbers in this group were too small to perform separate survival analysis). Eighteen percent (112) of 615 kidneys were not transplanted (Fig. 2).

**Table 1:** Outcome of 615 kidneys from 308 donors that were declined for paediatric recipients from 2009 to 2014 in the UK (46 paediatric kidney only transplants were from 43 donors)

Outcome of organ after decline for paediatric recipient	*N*	1-year patient survival			1-year renal allograft survival			3-year patient survival			3-year renal allograft survival		
		% Survival	95% CI		% Survival	95% CI		% Survival	95% CI		% Survival	95% CI	
Not transplanted	112												
Paediatric kidney only	46	97.7	84.6	99.7	89.1	75.8	95.3	97.7	84.6	99.7	82	67.1	90.6
Adult kidney only	384	95.7	92.7	97.5	96	93.5	97.6	93.1	89.1	95.6	93.9	90.7	96.1
Adult kidney and pancreas	61	100	–	–	96.7	87.5	99.2	97.9	85.8	99.7	87.4	73.5	94.3
Other	12^a^												

Patient and renal allograft survival data to 24 July 2016. ‘Other’ category includes double kidney (paediatric and adult), kidney and liver (paediatric and adult) and kidney and small bowel (adult). 4/12 kidneys went to children

*N* number, *CI* confidence interval

^a^Insufficient events in each group to be able to report survival

Of 485 kidneys from 243 donors declined due to donor reasons or size mismatch for paediatric recipients, 79% of these kidneys were eventually transplanted and 6% (28) of 485 went to paediatric kidney-only recipients (Table 2).

**Table 2:** Outcome of 485 kidneys from 243 donors that were declined due to donor reasons or size mismatch for paediatric recipients from 2009 to 2014 in the UK

Outcome of organ after decline for paediatric recipient	*N*	1-year patient survival			1-year renal allograft survival			3-year patient survival			3-year renal allograft survival		
		% Survival	95% CI		% Survival	95% CI		% Survival	95% CI		% Survival	95% CI	
Not transplanted	102												
Paediatric kidney only	28	96.3	76.5	99.5	82.1	62.3	92.1	96.3	76.5	99.5	78.6	58.4	89.8
Adult kidney only	308	95.1	91.5	97.2	95.7	92.7	97.5	92.2	87.9	95	93.7	90.1	96
Adult kidney and pancreas	40	100	–	–	97.5	83.5	99.6	100	–	–	85.9	69.2	93.9
Other	7^a^												

Patient and renal allograft survival data to 24 July 2016. ‘Other’ category includes double kidney (paediatric and adult), kidney and liver (paediatric and adult), and kidney and small bowel (adult). Two out of seven kidneys went to children

*N* number, *CI* confidence interval

^a^Insufficient events in each group to be able to report survival

### Outcome in paediatric patients for whom a kidney was offered and declined

**Fig. 3:**
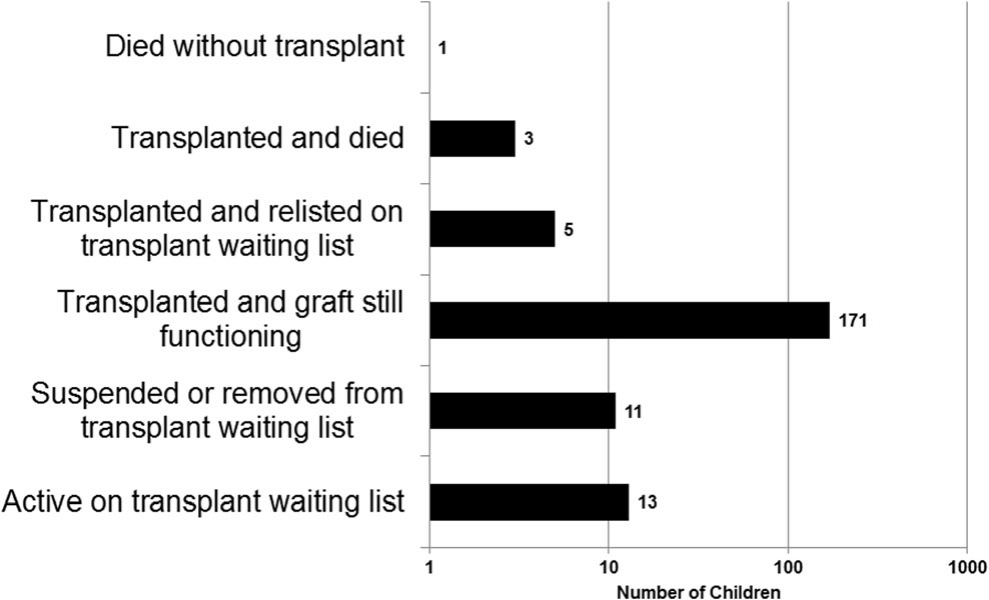
Patient outcomes for children who had a kidney offer declined on their behalf in the UK from 2009 to 2014 (logarithmic scale) outcomes taken after their first decline during this period (many patients had more than one declined offer and may have had declines outside this time period)

One child died while waiting for a transplant out of the 204 children who had a kidney offer declined on their behalf during the study. Eighty-four percent (171) of 204 children have been transplanted and have a functioning graft (Fig. 3).

The waiting time for a child to be transplanted after an offer was declined for them was 0 to 1701 (median 198) days (Table 3 for all children who received a transplant and Table 4 for those patients with initial kidney decline due to donor reasons or inappropriate size). These data consider the outcome following the patients first declined offer between 2009 and 2014. Many patients experienced more than one offer decline during this period and/or experienced declined offers outside of this time period. The data do not take account of any transplants prior to the decline offer of interest.

**Table 3:** Time between declined kidney offer of all children from 2009 to 2014 to transplantation

Donor type	*N*	Median time to transplant (days)	Range (days)
DBD	129	198	0–1701
DCD	8	152	55–445
LD	41	206	6–1093

*N* number, *DBD* donation after brain death, *DCD* donation after cardiac death, *LD* living donor

**Table 4:** Time between declined kidney offer due to donor reasons or size mismatch from 2009 to 2014 to transplantation

Donor type	*N*	Median time to transplant (days)	Range (days)
DBD	112	192	1–892
DCD	8	152	55–445
LD	37	186	6–1093

*N* number, *DBD* donation after brain death, *DCD* donation after cardiac death, *LD* living donor

### Prospective study of reasons for decline of a kidney for a paediatric recipient

There were 60 and 41 kidneys declined for paediatric recipients during the 2 years of prospective data collection (2011 and 2014, respectively). Although the absolute number of declines fell, the decline rate actually rose slightly from 59% in 2011 to 64% in 2014. This is because the number of children active on the kidney transplant waiting list fell from 101 in 2011 to 64 in 2014. One hundred percent of the additional supplemental data forms were returned from ten paediatric renal transplant centres in the UK.

In 75% of kidney declines for paediatric recipients, donor poor health or cause of death was cited as a reason for decline (Table 5). In order of importance, the three main donor health or cause of death reasons for decline were as follows: (1) death due to suspected meningoencephalitis without an identified organism, (2) virology (hepatitis B or C) or concern over donor substance misuse without virology results and (3) death from brain tumour without a histological diagnosis. There was no statistically significant difference between the 2 years in any of the reasons for decline (chi-squared test).

**Table 5:** Baseline data and reasons for decline for all kidneys declined on behalf of paediatric recipients in the UK from prospective audit data in 2011 and 2014

	2011	2014	Both Years
Total number of declines	60	41	101
Decline rate (based on total number on waiting list)	59%	64%	61%
Number of different donors	51	31	82
Number of different recipients	40	32	72
Donor age in years (median)	2–50 (38)	4–50 (40)	2–50 (40)
Recipient age in years (median)	1–17 (10)	2–17 (12)	1–17 (10)
Donor weight in kg (median)	14–180 (75)	16–137 (71)	14–180 (75)
Recipient weight in kg (median)	11–60 (20)	10–84 (32)	10–84 (26)
Donor height in cm (median)	91–200 (168)	117–189 (170)	91–200 (170)
Recipient height in cm (median)	76–178 (116)	71–171 (133)	71–178 (125)
Reasons for decline			
Donor poor health or cause of death	72%	80%	75%
Size mismatch	22%	15%	19%
Awaiting a better offer or live transplant	5%	10%	7%
Poor HLA match or positive cross match	8%	2%	6%
Recipient unfit for surgery	3%	2%	3%
Lack of staff to perform organ retrieval or transplant	3%	2%	3%
Ischaemia time (CIT or WIT) too long or organ damage during retrieval	3%	2%	3%
Other	3%	5%	4%

Some declined offers had multiple reasons for the decline; the percentages shown in the table reflect the proportion of declines where that reason applied

*HLA* human leukocyte antigen, *CIT* cold ischaemia time, *WIT* warm ischaemia time

## Discussion

Our findings show that kidneys declined for a paediatric recipient can have acceptable 1 and 3-year patient and renal allograft survival if eventually transplanted. Short-term renal allograft outcomes are better if these organs are transplanted into adult rather than paediatric recipients. The results also show that most children who have a kidney offer declined on their behalf will eventually be transplanted, but the median time to transplant is over 6 months. One child who initially had a kidney declined on their behalf died whilst waiting for a transplant. There are few studies in the literature reporting outcomes after declined kidney offers, and ours is the first to systematically assess organ declines for paediatric recipients across a national organ allocation network.

Whilst the observational nature of this study does not allow us to predict whether each child in this study would have benefitted from receiving the organ initially declined for them, the fact that 82% of 615 kidneys were eventually transplanted suggests that some of these kidneys were appropriate for paediatric transplantation, acknowledging different risk-benefit ratios in paediatric and adult renal transplant recipients. A kidney that is appropriate for an adult recipient is not necessarily appropriate for a paediatric recipient. The finding that 3-year renal allograft survival in the kidneys transplanted to adult recipients is acceptable cannot necessarily be applied to paediatric recipients. However, although most children with an initial decline were eventually transplanted, this study has demonstrated that declining a kidney offer for a child in the UK extends the transplant waiting time by a further 6 months on average. Whilst a child waiting an additional 6 months on an optimised form of renal replacement therapy (such as nocturnal home haemodialysis) may not suffer many additional risks, a child on sub-optimal dialysis treatment with other co-morbidities may suffer significant morbidity by waiting an additional 6 months for a transplant. Recent evidence has also shown that children not on dialysis at the time of transplant have improved renal allograft survival compared to those on dialysis at the time of transplant, although short periods of dialysis (< 6 months duration) did not confer any additional risk to renal allograft survival [8]. Therefore, the decision to refuse a kidney offer for a child must not be taken lightly, and better evidence is required to help clinicians make these difficult decisions.

As well as the observational nature of this study, a further limitation to the conclusions we can draw from these results is the small number of declined organs that eventually went to other paediatric recipients (46 in total). This has resulted in 1 and 3-year renal allograft survival estimates with wide confidence intervals and may in part account for the finding that 1-year renal allograft survival is better in adult recipients. A possible explanation for these results is that children who eventually accept a kidney that was initially declined for a paediatric recipient may be children who are difficult to transplant or have been waiting for a long time; therefore, the children that eventually get the kidney may have inferior renal allograft survival due to their individual recipient characteristics.

Once the 3-year renal allograft survival data is considered, it appears that adult recipients can have better outcomes than paediatric recipients when they get a kidney that was initially declined for a paediatric recipient. We cannot draw firm conclusions over the reasons for this, but the renal allograft survival estimate in our study does not differ significantly from renal allograft survival in larger cohorts of paediatric donation after brain death (DBD) kidney recipients. One-year renal allograft survival was 89% [95% CI 75.8–95.3%] for paediatric recipients, which is comparable to data from the UK transplant registry for 955 DBD kidney transplants for children where 1-year allograft survival was 93% [95% CI 91.6–94.8%; unpublished data: NHS Blood and Transplant]. Three-year renal allograft survival in the 46 kidneys that went to paediatric recipients in our study was 82% [95% CI 67.1–90.6%], which is also comparable to data from the UK transplant registry where 3-year allograft survival was 87% [95% CI 84.8–89%; unpublished data: NHS Blood and Transplant]. The difference in survival between patients in our study and the whole of the UK is not statistically significant, at both 1 and 3 years.

When we consider the 485 kidneys declined due to donor reasons or inappropriate size for paediatric recipients, only 28 of these were eventually transplanted to other paediatric recipients (Table 4). One-year renal allograft survival in this sub-group is 82% [95% CI 62.3–92.1%]. Although this does not differ significantly from the data for 955 DBD kidney transplants above, this is partly because the confidence intervals are wide due to small numbers. It is interesting to note that both 1 and 3-year renal allograft survival is worse for paediatric recipients than adult recipients, although the number in this sub-group is small and it is difficult to draw firm conclusions.

The prospective observational part of this study has enabled us to better understand the most common reasons for decline of a kidney offer for a paediatric recipient. Across the 2 years studied, 75% of declines cited poor donor health or cause of death as a reason for declining the organ. Nineteen percent of declines cite an inappropriate size match between donor and recipient as a reason for declining the organ.

The importance of good donor health is emphasised in the literature [6, 9], and this is particularly the case for paediatric recipients. Children with ESKD are likely to need multiple kidney transplants across their life, so maximising the longevity of the kidneys they receive during childhood is paramount [10]. However, there is emerging evidence from adult literature that suggests that inferior but acceptable transplant outcomes can be achieved with ‘marginal’ or ‘extended-criteria’ donors [6, 9]. Whilst this does not permit automatic extension of this evidence to paediatric recipients, when the negative impact of other forms of renal replacement therapy such as dialysis is considered [11], it may be prudent to consider widening the criteria of potential organ acceptance for paediatric recipients. We are already seeing increased utilisation of donation after DCD kidney transplants in both adults and children with good outcomes to date [12, 13], so there may be a case for extending the criteria for organ acceptance for paediatric recipients.

The issue of size mismatch is a controversial one in paediatric renal transplantation. Whilst the potential surgical and haemodynamic problems of large kidneys for small recipients are well documented [7], there are reports of similar renal allograft outcomes to size-matched grafts [14] and these concerns must be balanced against the well-recognised risks of staying on dialysis in such young patients [11]. This is even more so the case in this situation, where a size mismatch is also likely to occur for any potential living-related donor, and may also prevent this type of transplantation occurring. Size mismatch is not listed as a contraindication in the NHS Blood and Transplant guideline (2013) outlining reasons for decline of kidneys for paediatric recipients [15]. This is generally a decision made at a local level by the recipient’s individual nephrologist and transplant surgeon, where size mismatch may eventually be a factor which results in a decision to decline by the operating surgeon.

Although this part of our study helps us to understand the commonest reasons for decline of a kidney offer for a paediatric recipient, further study is required to help clarify these factors and to provide evidence for guideline development. In particular, detailed analysis of which donor health issues preclude transplant and the specifics of size mismatch (e.g. kidney too big or small) will help to evaluate these factors further.

In conclusion, this study demonstrates that most kidneys initially declined for paediatric transplantation can be successfully transplanted into both adult and paediatric recipients, with acceptable 1 and 3-year patient and renal allograft survival (although there may be an advantage for adult renal transplant recipients).

The decision to decline a kidney for a paediatric recipient must not be taken lightly, as our results demonstrate that children will wait an additional 6 months on average, to receive a transplant. Robust evidence-based guidelines are required to aid clinical decision making in order to ensure that the most appropriate kidneys are selected for paediatric recipients.
